# *FGFR2* is a Crucial Factor for Adipose‐Derived Mesenchymal Stem Cells in Promoting Diabetic Foot Ulcer Healing Through Angiogenesis

**DOI:** 10.1111/jcmm.70942

**Published:** 2025-11-13

**Authors:** Jing Cao, Zichao Liu, Wenqiang An, Xin Zhang, Zhujun Li, Lijie Li, Hailian Ji, Sen Zhang, Xiao Long, Yuemei Yang

**Affiliations:** ^1^ Beijing AegleStem Therapeutics Co., Ltd Beijing China; ^2^ Department of Plastic & Cosmetic Surgery Peking Union Medical College Hospital Beijing China; ^3^ Institute of Materia Medica Chinese Academy of Medical Sciences & Peking Union Medical College Beijing China

**Keywords:** adipose‐derived mesenchymal stem cells, angiogenesis, diabetic foot ulcers, *FGFR2*

## Abstract

Diabetic foot ulcers (DFUs) remain a significant clinical challenge due to the lack of effective treatments, severely impacting patients' quality of life. Mesenchymal stem cells (MSCs) have shown potential in promoting DFU healing; however, the underlying mechanisms are not yet fully understood. This study investigates the role of adipose‐derived mesenchymal stem cells (ADSCs) in DFU healing, with a particular focus on angiogenesis. Gene expression profiles from the GSE7014 and GSE80178 datasets in the Gene Expression Omnibus (GEO) database were analyzed. Differentially expressed genes (DEGs) were intersected with angiogenesis‐related genes from the GeneCards database, identifying 35 angiogenesis‐related DEGs (An‐DEGs). Key genes were selected using Cytoscape software and machine learning. The pro‐angiogenic effects of ADSCs were validated through in vivo and in vitro experiments, assessing their role in DFU healing. The DEGs from DFU patients were enriched in pathways such as angiogenesis and collagen‐containing extracellular matrix. ADSCs promoted angiogenesis and wound healing by upregulating *FGFR2* and secreting FGF, activating the FGF‐PI3K/Akt‐HIF‐1α‐VEGF axis. Additionally, ADSCs mediated secretion of VEGF concerting this effect. *FGFR2* plays a pivotal role in ADSCs' mediated DFU healing by driving angiogenesis.

## 1 Introduction

Diabetic foot ulcers (DFUs) are one of the most prevalent complications of diabetes, with a lifetime risk of up to 25% in individuals with diabetes [1, 2]. The development of DFUs is multifactorial, involving sensory loss, ischemia, and trauma [3]. Diabetic peripheral neuropathy and peripheral vascular disease interact synergistically, exacerbating the formation and progression of DFUs [4]. Among DFU patients, 15%–27% will require amputations to varying degrees [2, 5]. The five‐year survival rate following amputation is less than 30%, much lower than the overall five‐year survival rate of 68% for cancer, severely affecting the survival rate of patients [1, 6]. Given these dire statistics, exploring novel therapeutic interventions with translational potential is of paramount importance, benefiting both individual patients and broader public health efforts [7, 8].

Angiogenesis, the formation of new blood vessels from the existing vascular network, plays a pivotal role in wound healing [9]. This process involves endothelial cell proliferation, migration, and tubular structure formation, all of which are regulated by various growth factors and cytokines [10]. However, patients with diabetes exhibit compromised angiogenic potential; this severely impairs wound healing [7, 11]. Persistent hyperglycemia impairs endothelial cell metabolism, leading to a series of microcirculation dysfunctions, including the downregulation of growth factors such as VEGF and FGF, ultimately disrupting angiogenesis. Moreover, reduced responsiveness of endothelial cells to pro‐angiogenic signals leads to diminished peripheral blood flow and inadequate local vascularization, contributing to chronic, non‐healing DFUs [7]. Prior studies in mouse models with diabetes have demonstrated that insufficient VEGF secretion impairs endothelial nitric oxide synthase (eNOS) phosphorylation in bone marrow, thereby hindering the mobilisation of endothelial progenitor cells (EPCs) into circulation [10]. Meanwhile, other studies indicated that the reduced expression of stromal cell‐derived factor 1α (SDF‐1α) in epithelial cells and myofibroblasts in the diabetic wound site prevents EPCs from homing to the wound [7, 12]. Consequently, enhancing local angiogenesis represents a key therapeutic strategy for DFUs.

Adipose‐derived mesenchymal stem cells (ADSCs) have emerged as a promising therapeutic approach for DFUs due to their immunomodulatory properties, tissue regenerative potential, and secretion of bioactive factors [13, 14, 15]. Recent studies have shown that ADSCs can enhance angiogenesis, improve blood perfusion, and accelerate tissue regeneration in wound sites [11, 16]. For instance, exosomes derived from ADSCs have been shown to accelerate angiogenesis in wound healing via the EGR‐1/lncRNA‐SENCR/DKC1/VEGF‐A axis [17]. Similarly, exosomes from human urine‐derived mesenchymal stem cells have also been reported to enhance angiogenesis and facilitate DFU healing [18]. Additionally, ADSCs can secrete growth factors such as VEGF, which is essential for promoting wound healing [19].

Fibroblast growth factor receptor 2 (*FGFR2*), a principal member of the *FGFR* family, mediates cellular responses to FGF ligands and is well established as a regulator of proliferation, migration, differentiation, and survival in multiple cell types [20, 21, 22]. Importantly, FGFR2 signalling has been implicated directly in vascular biology: activation of the FGF–FGFR2 axis promotes endothelial cell proliferation and migration, enhances capillary‐like tube formation, and contributes to vessel remodelling during tissue repair [23, 24, 25, 26]. Downstream effectors of *FGFR2* include the PI3K/Akt pathway and HIF‐1α‐related responses, both of which are central to angiogenic programs and to the cellular adaptation to hypoxia in wounded tissue [27]. Moreover, recent preclinical work showed that pharmacologic activation of *FGFR* (e.g., with *FGFR* agonist peptides) can accelerate angiogenesis and collagen deposition in diabetic wound models, supporting a potentially actionable role for *FGFR2* in impaired diabetic healing [28].

Although adipose‐derived stem cells (ADSCs) have been repeatedly shown to promote angiogenesis and improve healing in diabetic wounds—through paracrine secretion of growth factors (e.g., VEGF, FGF2), immunomodulation, and release of extracellular vesicles/exosomes and proteins [29, 30, 31]. It remains unclear whether ADSCs exert their pro‐angiogenic effects in DFUs by directly modulating FGFR2 signalling. Clarifying whether ADSCs activate FGFR2‐mediated pathways to facilitate DFU healing is therefore essential to delineate the molecular basis of ADSCs therapy and to identify targeted strategies that may potentiate angiogenesis and therapeutic translation in DFU management.

**FIGURE 1:**
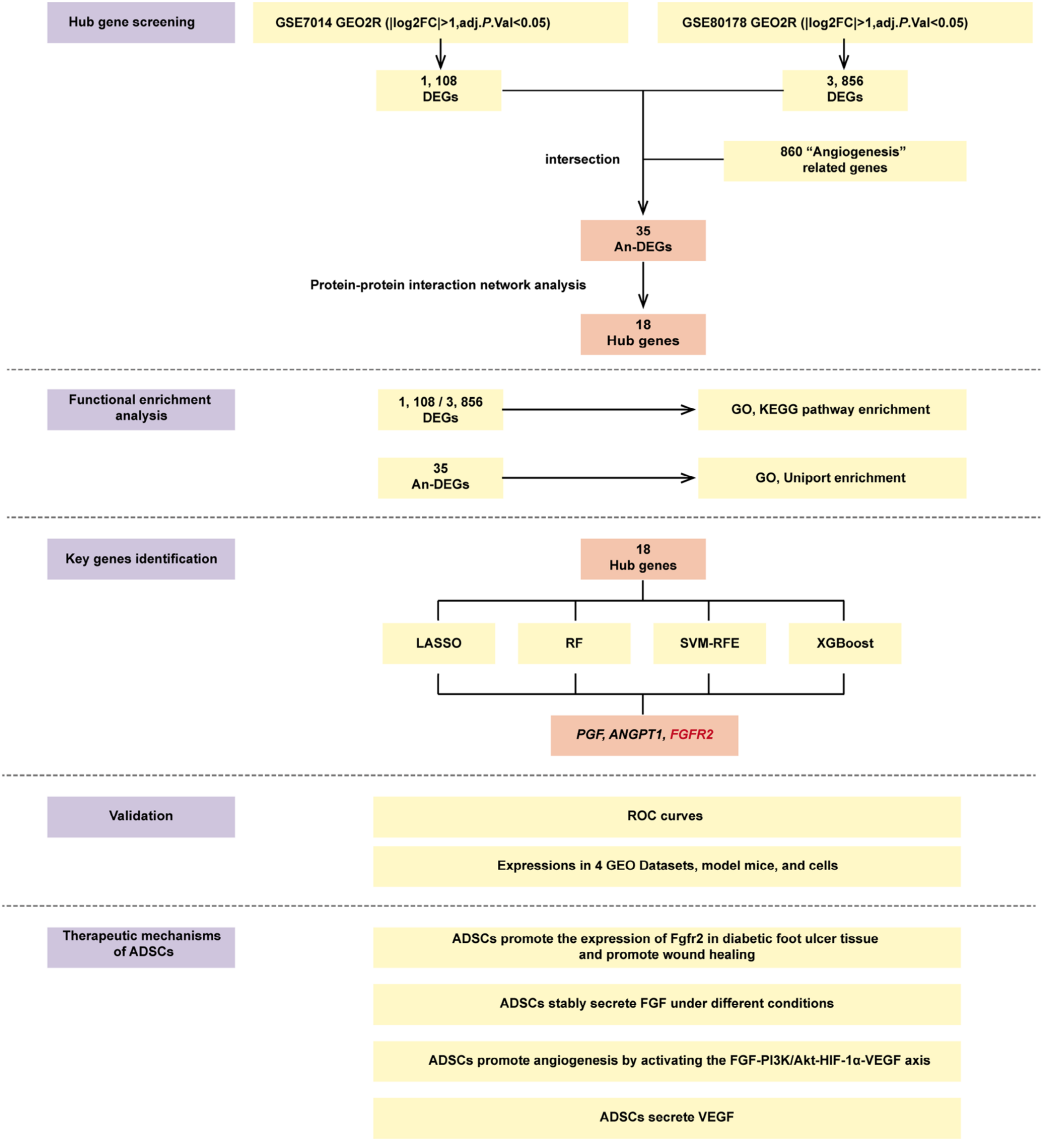
The flow chart of this study.

In this study, we analysed two publicly available datasets, GSE7014 and GSE80178, and found that differentially expressed genes (DEGs) were enriched in signalling pathways such as angiogenesis, PI3K, HIF‐1α, and extracellular matrix. By intersecting the DEGs from these two datasets with angiogenesis‐related genes, we identified 35 An‐DEGs. Using a protein–protein interaction (PPI) network, we ranked key genes based on their importance and further refined our selection through four distinct machine learning algorithms, ultimately identifying three key angiogenesis‐related genes. Finally, in vivo experiments in a mouse model with diabetes and in vitro studies using human umbilical vein endothelial cells (HUVECs) validated that ADSCs enhance the *FGFR2* expression and secrete FGF, thereby activating the FGF‐PI3K/Akt‐HIF‐1α‐VEGF signalling cascade, which promotes angiogenesis and facilitates DFU healing. At the same time, ADSCs can also directly secrete VEGF to synergize with this regulatory effect. This multifactorial and synergistic mechanism suggests that ADSCs may exert a more potent therapeutic effect than the administration of growth factors alone. The flow chart of this study is summarised in (Figure 1).

## 2 Material and Methods

### 2.1 Identification of Differentially Expressed Genes

DFU‐related datasets, GSE7014 and GSE80178, were retrieved from the GEO database (https://www.ncbi.nlm.nih.gov/geo/). Detailed information of the datasets is shown in Table 1. Differential expression analysis was carried out using GEO2R (https://www.ncbi.nlm.nih.gov/geo/geo2r/). Genes with an absolute log2 fold change (|log2FC|) > 1 and an adjusted *p* value < 0.05 were identified as DEGs and used for subsequent functional enrichment analysis.

**TABLE 1:** Information of the datasets.

Dataset	Platform	Count	Diabetes	Control
GSE80178	GPL16686	9	6	3
GSE7014	GPL570	36	30	6
GSE134431	GPL18573	21	13	8
GSE199939	GPL24676	22	10	11

### 2.2 Functional Enrichment Analysis

Gene Ontology (GO) and Kyoto Encyclopedia of Genes and Genomes (KEGG) pathway analyses were performed using the ‘clusterProfiler’ [32] package in R (version 4.4.1) software (https://www.r‐project.org/). The human genome was annotated using the ‘org.Hs.eg.db’ package. The results of the analysis were visualised using the ‘ggplot2’ package.

### 2.3 Gene Set Enrichment Analysis (GSEA)

GSEA analysis was performed using GSEA v4.3.3 software (http://www.gsea‐msigdb.org/gsea/downloads.jsp). The ‘GseaVis’ package in R was used for visualisation.

### 2.4 Identification of Hub Genes

The DEGs identified from the above differential expression analysis were intersected with ‘Angiogenesis’‐related genes from GeneCards (filtering for genes with a Relevance score ≥ 2, https://www.genecards.org/) to extract An‐DEGs. A PPI network was constructed by importing An‐DEGs into the STRING database (https://string‐db.org/) and Cytoscape software (version 3.10.3, https://cytoscape.org/). We included interactions with confidence scores of 0.4 or higher, corresponding to a medium confidence level. Gene importance was ranked using four algorithms—Maximum Neighbourhood Component (MNC), Maximal Clique Centrality (MCC), Degree, and Edge Percolated Component (EPC)—from the cytoHubba plugin in Cytoscape. MNC highlights genes located in densely connected functional modules, reflecting local coherence. MCC captures central elements of highly interconnected protein complexes. EPC reduces noise by selecting interactions with higher STRING confidence scores (≥ 0.4), thus improving reliability. Degree identifies nodes with extensive connectivity, representing their global influence within the network. The top 20 genes identified by each method were intersected to determine the hub genes.

### 2.5 Machine Learning‐Based Feature Selection

Feature selection was performed using four machine learning algorithms: Least Absolute Shrinkage and Selection Operator (LASSO), Support Vector Machine Recursive Feature Elimination (SVM‐RFE), Random Forest, and eXtreme Gradient Boosting (XGBoost). These algorithms were implemented using the R packages ‘glmnet’, ‘e1071’, ‘randomForest’, and ‘xgboost’. The analysis results were visualised using the ‘ggplot2’ package.

### 2.6 Establishment of a DFU Mouse Model and ADSCs Administration

Male BALB/c mice (6–8 weeks old, 18–20 g), were purchased from Sipeifu (Beijing) Biotechnology Co. Ltd. to establish a DFU mouse model. The DFU model was established using streptozotocin (STZ). Mice were fasted for 12 h before STZ injection (120 mg/kg, intraperitoneally) to induce diabetes. Mice with fasting blood glucose levels > 11.1 mmol/L were considered diabetic. Under anaesthesia and sterile conditions, a 4 mm full‐thickness wound was created on the dorsal skin using a biopsy punch.

The mice were randomly assigned into three groups (*n* = 7 per group) using the Excel *RAND* function. The experimental group received subcutaneous injections of 100 μL of ADSCs (25 μL/site) around the wound. The model group and non‐diabetic negative control group were injected with the same volume of cell buffer.

All animal experiments were conducted in compliance with the ARRIVE guidelines and in accordance with the National Research Council's Guide for the Care and Use of Laboratory Animals. Ethical approval for these experiments was granted by the Ethics Committee of Chinese Academy of Medical Sciences.

### 2.7 Immunohistochemistry

To assess angiogenesis, immunohistochemistry (IHC) was performed for CD31 (an endothelial cell marker) and VEGF. Tissue samples were fixed in formalin and embedded in paraffin. Paraffin‐embedded sections (5 μm) were deparaffinised and subjected to antigen retrieval using citrate buffer (pH 6.0). Sections were incubated with primary antibodies (1:200 dilution) overnight at 4°C, followed by incubation with a secondary antibody. Diaminobenzidine (DAB) was used for colour development, and sections were counterstained with haematoxylin. Microscopic evaluation was conducted to quantify CD31+ vessels and VEGF optical density. Statistical comparisons were made between the treated and control groups to evaluate therapeutic efficacy.

### 2.8 Western Blot

Proteins were extracted using radioimmunoprecipitation assay (RIPA) buffer supplemented with phenylmethylsulfonyl fluoride (PMSF, 1:100). Protein concentration was measured using a BCA kit, and 20 μg of protein was loaded for electrophoresis. β‐actin or Gapdh was used as the internal control.

### 2.9 Reverse Transcription Quantitative PCR (RT‐qPCR)

Total RNA was extracted from cells and mouse tissues using TIANGEN RNA extraction kits (DP430 and DP451, Beijing, China). Reverse transcription was performed using the TIANGEN FastKing DNA Dispelling RT SuperMix kit (KR118‐02, Beijing, China). RT‐qPCR was performed using the TaKaRa kit (RR820A, Dalian, China). GAPDH was used as the internal control. Relative mRNA expression levels were calculated using the 2^−ΔΔCT^ method. Primer sequences are listed in Table 2.

**TABLE 2:** Primer sequences.

Human *FGFR2*	Forward:	ATGCCCGTAGAGGAAGTGTG
	Reverse:	TGGTATTTGGTTGGTGGCTC
Human *GAPDH*	Forward:	ACGGATTTGGTCGTATTGGG
	Reverse:	GGGATCTCGCTCCTGGAAG
Mouse Fgfr2	Forward:	CAAAGGCAACTACACCTGC
	Reverse:	GAAGTCTGGCTTCTTGGTCG
Mouse Gapdh	Forward:	CATGGCCTTCCGTGTTCCTA
	Reverse:	TGTCATCATACTTGGCAGGTTTCT

### 2.10 3D Tube Formation Assay

Sixty microlitre of Matrigel was added to each well of a 96‐well plate (on ice), and the plate was incubated at 37°C for 30 min to solidify the Matrigel. HUVECs were seeded into the precoated 96‐well plate at a density of 5000 cells per well. The cells were cultured at 37°C with 5% CO_2_, and the formation of tubular structures in HUVECs was observed and photographed at the 4th and 6th hour using a phase contrast microscope. For HUVECs cultured in an advanced glycation end‐products (AGEs) environment (AGE–HUVECs), AGEs were purchased from ChemeGen (Cat. No. CY30824). A stock solution was prepared by dissolving 10 mg of AGEs in PBS to yield a 10 mg/mL solution, which was then diluted 1:50 in culture medium to obtain a 200 μg/mL working concentration. AGEs were added at the time of cell seeding and maintained throughout the entire culture period. For the ADSCs co‐culture group, equal numbers of passage 5 (P5) ADSCs were seeded simultaneously. The PI3K signalling pathway was inhibited using PI3K‐IN (MedChemExpress, Cat. No. HY‐12068).

### 2.11 Harvest of Conditioned Medium From ADSCs (CM)

P5 ADSCs were seeded in T75 culture flasks, with approximately 6 × 10^5^ cells per flask, and cultured in serum‐free medium. After 72 h, the culture supernatant was collected and centrifuged at 300 *g* to remove cells and cell debris.

### 2.12 Migration Assay

HUVECs were seeded at a density of 4 × 10^5^ cells per well in a 6‐well plate. The cells were cultured in 2 mL of endothelial cell medium (ECM) complete medium per well for 6 h until attachment, followed by overnight serum starvation. The next day, a 0.2 mL sterile pipette tip was used to create scratches before replacing the medium with the corresponding culture medium for each group. With FGF neutralising antibody (Ab1), VEGF neutralising antibody (Ab2) at a concentration of 5 ng/mL, the groups were HUVECs, HUVECs + CM, HUVECs + CM + Ab1, HUVECs + CM + Ab1 + Ab2. The scratch wounds were observed and photographed under a microscope at 0, 6, and 24 h post‐scratching to assess cell migration.

### 2.13 Statistical Analysis

Data were analysed using GraphPad Prism 9 or R 4.4.1, and presented as mean ± standard deviation (SD). Group comparisons were conducted using Student's *t*‐test or one‐way ANOVA. *p* value < 0.05 was considered statistically significant. *p < 0.05, **p < 0.01, ***p < 0.001, and ****p < 0.0001.

## 3 Results

### 3.1 Transcriptome Analysis

**FIGURE 2:**
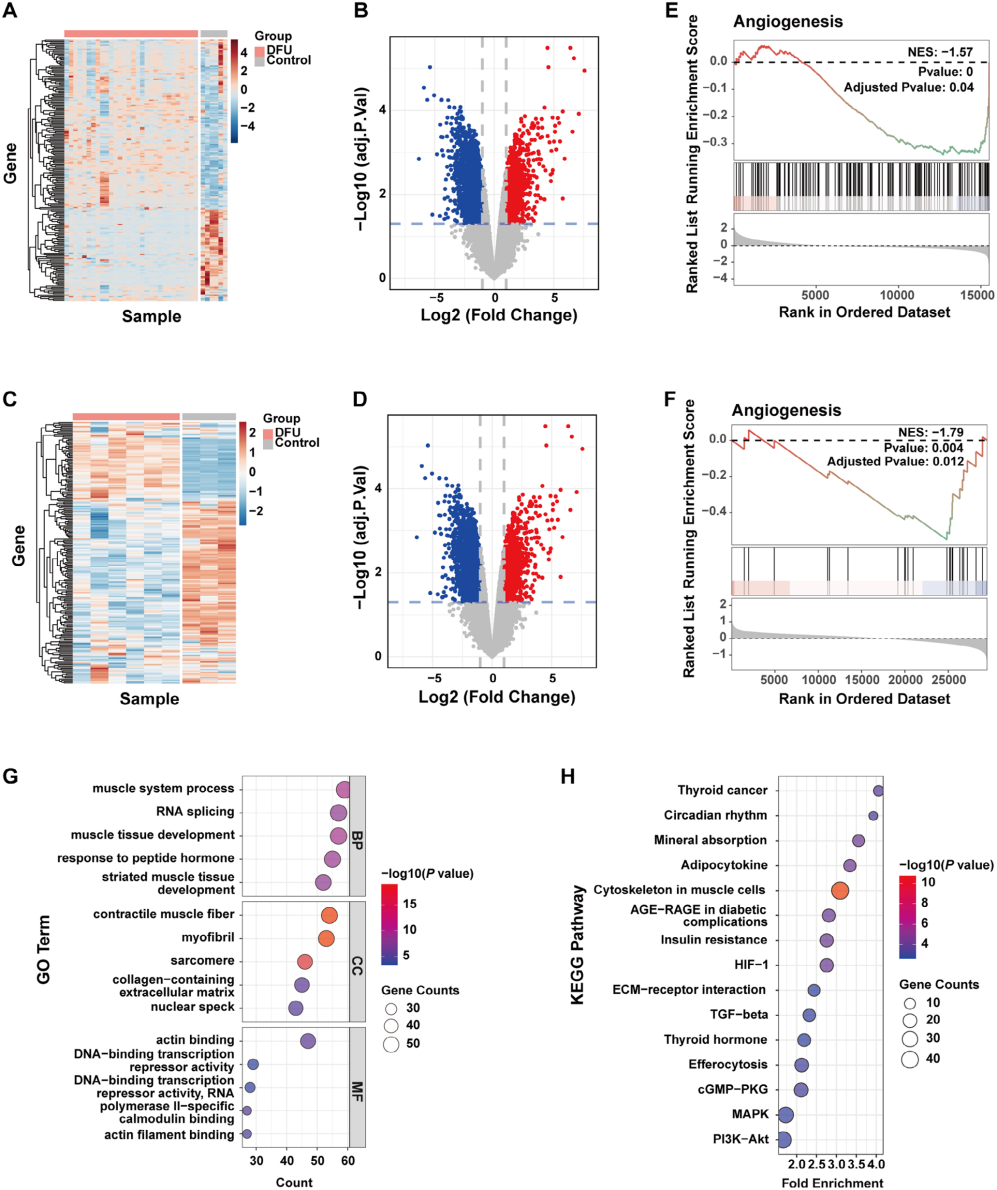
Transcriptome analysis. (A) Heatmap and (B) Volcano plots of DEGs in dataset GSE7014. (C) Heatmap and (D) Volcano plots of DEGs in dataset GSE80178. (E and F) GSEA analysis results of angiogenesis in GSE7014 and GSE80178. (G) GO and (H) KEGG pathway analysis in GSE7014.

To investigate the molecular mechanism underlying the development of DFUs, we first performed differential expression analysis. A total of 1107 and 3856 DEGs were identified in DFU and control patients from the GSE7014 and GSE80178 datasets, respectively (Figure 2A–D). GSEA revealed a significant suppression of angiogenesis‐related pathways in patients with diabetes across both datasets (Figure 2E,F). Further functional enrichment analysis indicated that DEGs were primarily enriched in pathways related to the extracellular matrix (collagen‐containing in extracellular matrix), skin development, HIF‐1α signalling, AGE‐RAGE signalling in diabetic complications, and PI3K signalling (Figure 2G,H, Figure S1A,B); the full list of enriched GO terms and KEGG pathways (adjusted *p* < 0.05) is now provided in Tables S1 and S2.

### 3.2 Identification of 18 Hub Genes via PPI Network Analysis

**FIGURE 3:**
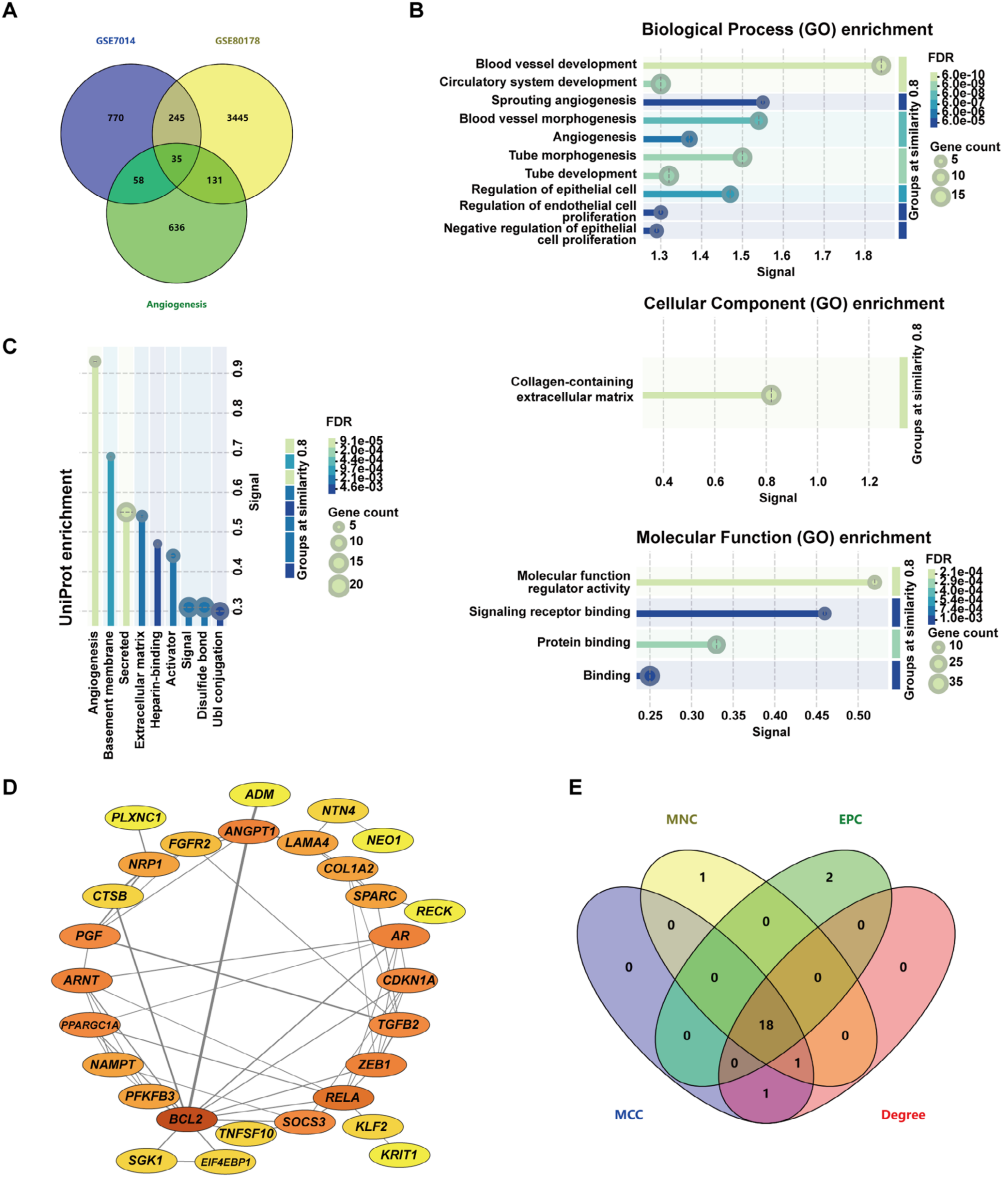
Screening of hub genes. (A) Venn diagram of the intersection of angiogenesis‐related genes and DEGs in GSE7014 and GSE80178. (B and C) GO and UniProt enrichment analysis of An‐DEGs. (D) PPI network of An‐DEGs. (E) Venn diagram showing the intersection of the top 20 genes ranked by four algorithms in CytoHubba.

In order to determine the angiogenesis‐related genes implicated in DFU pathogenesis, we intersected the DEGs identified in GSE7014 and GSE80178 with angiogenesis‐related genes in GeneCards, yielding 35 An‐DEGs (Figure 3A). The 35 An‐DEGs were imported into the STRING database for molecular interaction analysis. GO analysis revealed that these 35 An‐DEGs were significantly enriched in pathways related to angiogenesis and blood vessel development (Figure 3B). Similarly, UniProt enrichment analysis also confirmed their association with angiogenesis and extracellular matrix (Figure 3C). The PPI network for the An‐DEGs was constructed using the STRING database and visualised in Cytoscape (Figure 3D). To identify key regulatory genes, four ranking algorithms (MNC, MCC, Degree, and EPC) from the CytoHubba plugin of Cytoscape were applied. The intersection of the top 20 genes from each algorithm resulted in 18 hub genes (Figure 3E).

### 3.3 *FGFR2* Is a Key Factor in DFU Pathogenesis

**FIGURE 4:**
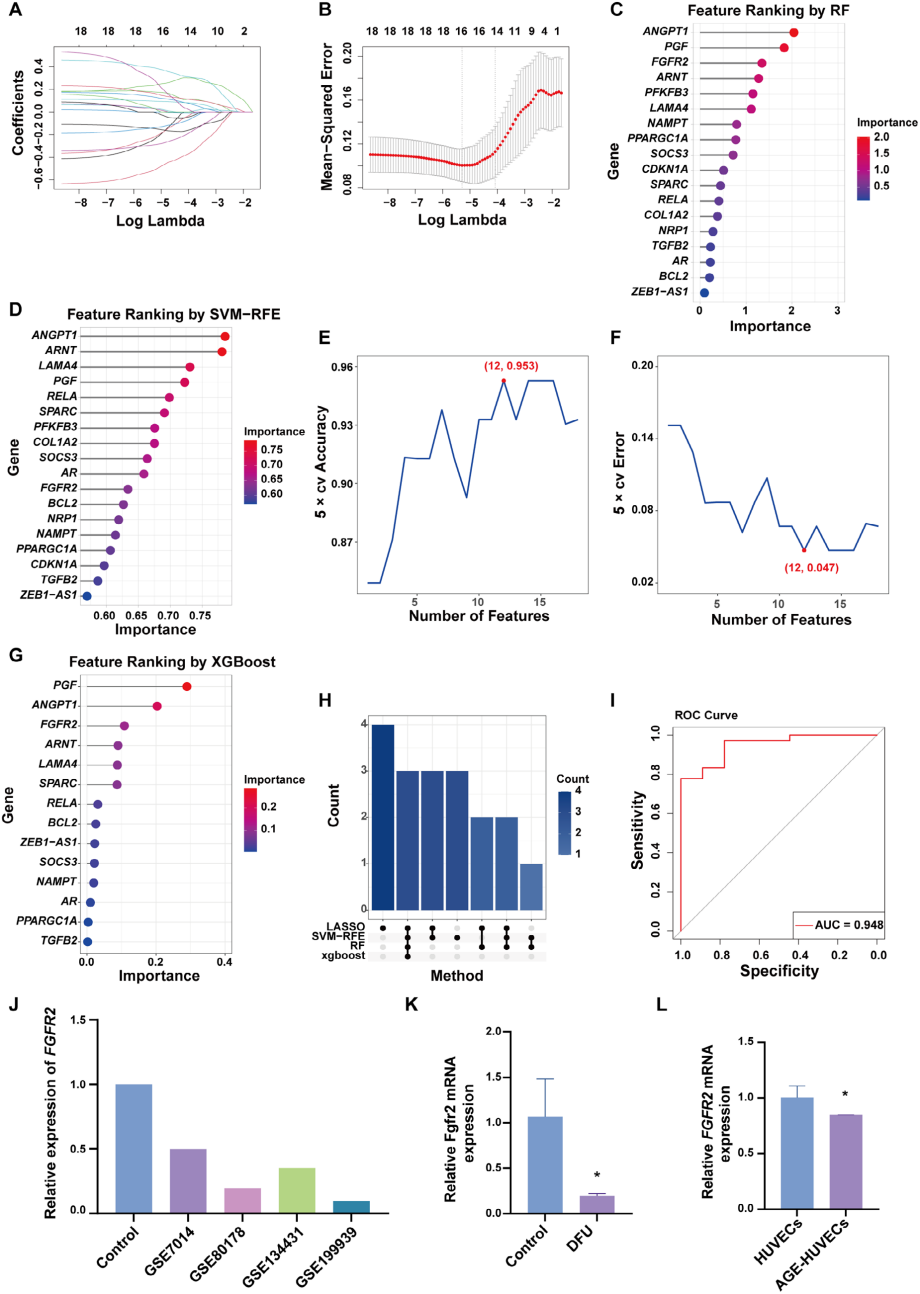
Machine learning‐based identification of key angiogenesis‐related genes in DFUs. (A and B) Path plot and cross‐validation results of LASSO regression. (C) Gene importance ranking plot from the RF algorithm. (D–F) Gene importance ranking and cross‐validation accuracy and error results from the SVM‐RFE algorithm. (G) Gene importance ranking from the XGBoost algorithm. (H) Upset plot showing the distribution of genes selected by the four algorithms. (I) ROC curve of the three selected genes. (J) Relative expression of *FGFR2* in the DFU patient groups from datasets GSE7014, GSE80178, GSE134431, and GSE199939. (K) *FGFR2* expression in the DFU mouse model. (L) *FGFR2* expression in AGE‐HUVECs. *p < 0.05.

To further narrow down the key factors in the development and progression of DFUs, we performed feature selection using four machine learning algorithms: LASSO, RF, SVM‐RFE, and XGBoost. Batch correction and normalisation of the data from datasets GSE7014 and GSE80178 were performed using the R packages ‘sva’ and ‘limma’ (Figure S2A,B). The two datasets were then merged to form the training set for machine learning. LASSO regression identified 16 key genes (Figure 4A,B), RF identified 8 (Figure 4C), SVM‐RFE identified 12 (Figure 4D–F), and XGBoost identified 3 (Figure 4G). The intersection of these four methods revealed three key genes: *PGF*, *ANGPT1*, and *FGFR2* (Figure 4H). Receiver operating characteristic (ROC) curve analysis demonstrated that these genes exhibited high predictive value for DFUs, with an area under the curve (AUC) of 0.948 (Figure 4I). Further analysis showed that *FGFR2* expression was significantly downregulated in the DFU patient groups across multiple datasets (GSE7014, GSE80178, GSE134431, and GSE199939) (Figure 4J). Subsequent experimental validation confirmed that *FGFR2* was also downregulated in DFU mice and AGEs‐treated HUVECs (Figure 4K,L). Therefore, through machine learning and experimental validation, we identified *FGFR2* as a critical factor in DFU pathogenesis.

### 3.4 ADSCs Enhance Fgfr2 Expression in DFUs and Promote DFUs Wound Healing

**FIGURE 5:**
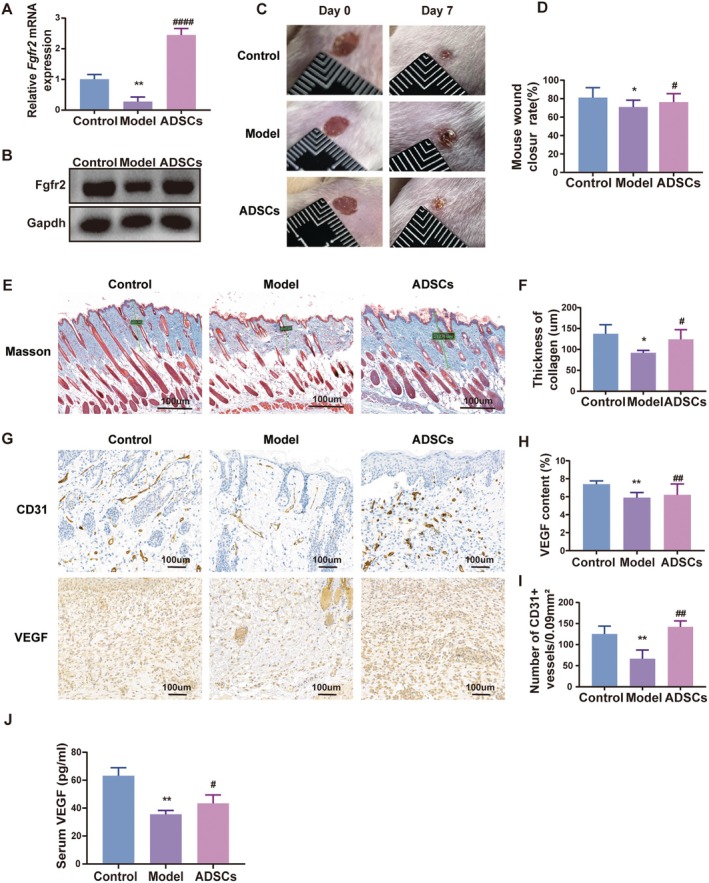
ADSCs enhance Fgfr2 expression in DFUs and promote DFUs wound healing. (A and B) Fgfr2 is upregulated in the ADSCs treated DFU tissues. (C and D) ADSCs improve the wound healing rate in DFU model mice. (E and F) Masson's trichrome staining of skin tissues in DFU model mice before and after ADSCs treatment. (G–I) Quantification of neovascularization and VEGF levels in skin tissues of DFU model mice before and after ADSCs treatment. (J) Serum VEGF levels in DFU mice before and after ADSCs treatment. *p < 0.05, **p < 0.01 vs. Control; ^#^ *p* < 0.05, ^##^ *p* < 0.01, ^####^ *p* < 0.0001 vs. Model, *n* = 7. Scale bar: 100 μm.

To elucidate the therapeutic mechanism of ADSCs in DFUS wound healing, we assessed Fgfr2 expression following ADSCs administration in DFU mice. RT‐qPCR results showed that Fgfr2 was significantly upregulated in DFU tissues after ADSCs treatment (Figure 5A,B). Macroscopic images of mouse wounds revealed that the wound healing rate in DFU mice was significantly increased after ADSCs treatment (Figure 5C,D). Masson's trichrome staining showed a significant increase in collagen deposition in the dermal layer of DFU mice following ADSCs treatment (Figure 5E,F). IHC results indicated that ADSCs treatment significantly increased the number of newly formed blood vessels per unit area and elevated VEGF levels in the skin tissue in DFUs wound (Figure 5G–I). Additionally, ELISA analysis of mouse serum showed a significant increase in VEGF content after ADSCs treatment (Figure 5J). The above results indicate that after ADSCs treatment, Fgfr2 is significantly elevated in DFU tissues, and ADSCs may promote wound healing by increasing VEGF levels in DFU tissues and serum through the upregulation of Fgfr2 expression.

### 3.5 ADSCs Activate the FGF‐PI3K/Akt‐HIF‐1α‐VEGF Axis to Promote Angiogenesis

**FIGURE 6:**
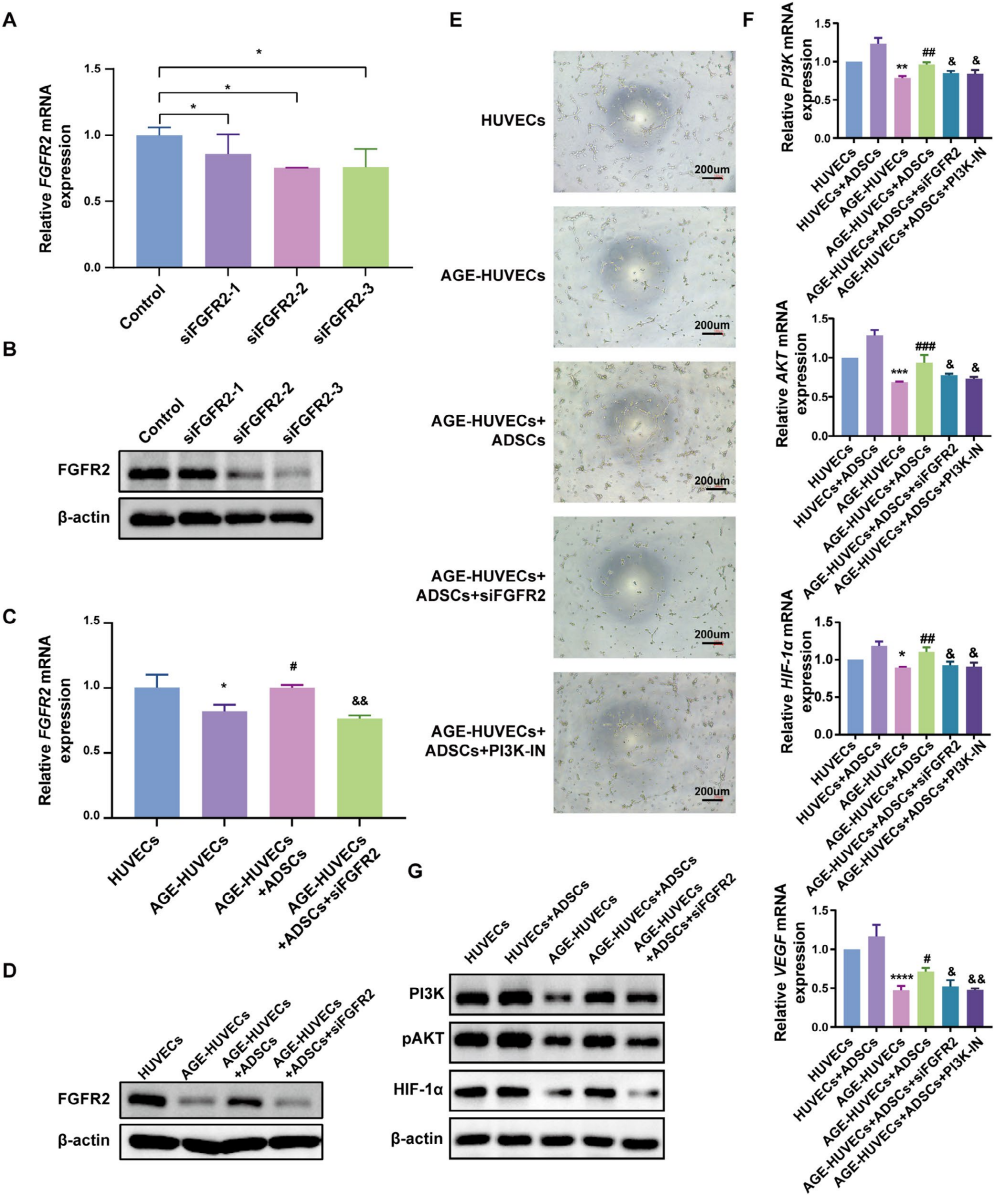
ADSCs activate the FGF‐PI3K/Akt‐HIF‐1α‐VEGF axis to promote angiogenesis. (A and B) RT‐qPCR and Western blot confirming the knockdown efficiency of siFGFR2 on *FGFR2* expression. *p < 0.05. (C and D) RT‐qPCR and Western blot analysis showing that siFGFR2 inhibits ADSCs‐induced *FGFR2* upregulation. (E) siFGFR2 and PI3K inhibitor (PI3K‐IN) inhibit the pro‐angiogenic effect of ADSCs. (F) siFGFR2 inhibits the expression of *PI3K* and *AKT* in HUVECs, while PI3K‐IN suppresses *HIF‐1α* and *VEGF* expression. *p < 0.05, **p < 0.01, ***p < 0.001, ****p < 0.0001 vs. HUVECs; ^#^ *p* < 0.05, ^##^ *p* < 0.01, ^###^ *p* < 0.001 vs. AGE‐HUVECs; ^&^ *p* < 0.05, ^&&^ *p* < 0.01 vs. AGE‐HUVECs + ADSCs. (G) siFGFR2 inhibits the expression of PI3K, pAKT and HIF‐1α in HUVECs. Scale bar: 200 μm.

To further investigate the molecular mechanism by which ADSCs promote wound healing through regulation of *FGFR2* expression, we examined the downstream signalling axis of *FGFR2* in HUVECs based on previous bioinformatics analysis. First, *FGFR2* expression in HUVECs was knocked down using siRNA, and as shown in the figure, siRNA‐2 and siRNA‐3 showed good knockdown efficiency and siRNA‐3 was used in subsequent experiments (Figure 6A,B). Further experiments revealed that co‐culture of ADSCs with AGE‐HUVECs effectively increased the expression of *FGFR2* in AGE‐HUVECs. Whereas this effect was abolished in *FGFR2* silenced AGE‐HUVECs (Figure 6C,D). Tube formation assays showed that the angiogenic capability of AGE‐HUVECs was significantly weaker than that of untreated HUVECs. ADSCs co‐culture significantly enhanced angiogenesis in AGE‐HUVECs. However, this effect was suppressed by either *FGFR2* knockdown or PI3K inhibition (Cat. No. HY‐12068, MCE) (Figure 6E). Therefore, it is speculated that ADSCs promote angiogenesis by activating the FGF and PI3K signalling pathways. Subsequently, RT‐qPCR analysis and Western blot showed that knockdown of FGFR2 led to a reduction in *PI3K* and *AKT* expression, and inhibition of PI3K suppressed *HIF‐1α* and *VEGF* gene expression (Figure 6F,G). These findings suggest that ADSCs promote angiogenesis via activation of the FGF‐PI3K/Akt‐HIF‐1α‐VEGF axis.

### 3.6 ADSCs Secrete Cytokines to Synergistically Promote Angiogenesis

**FIGURE 7:**
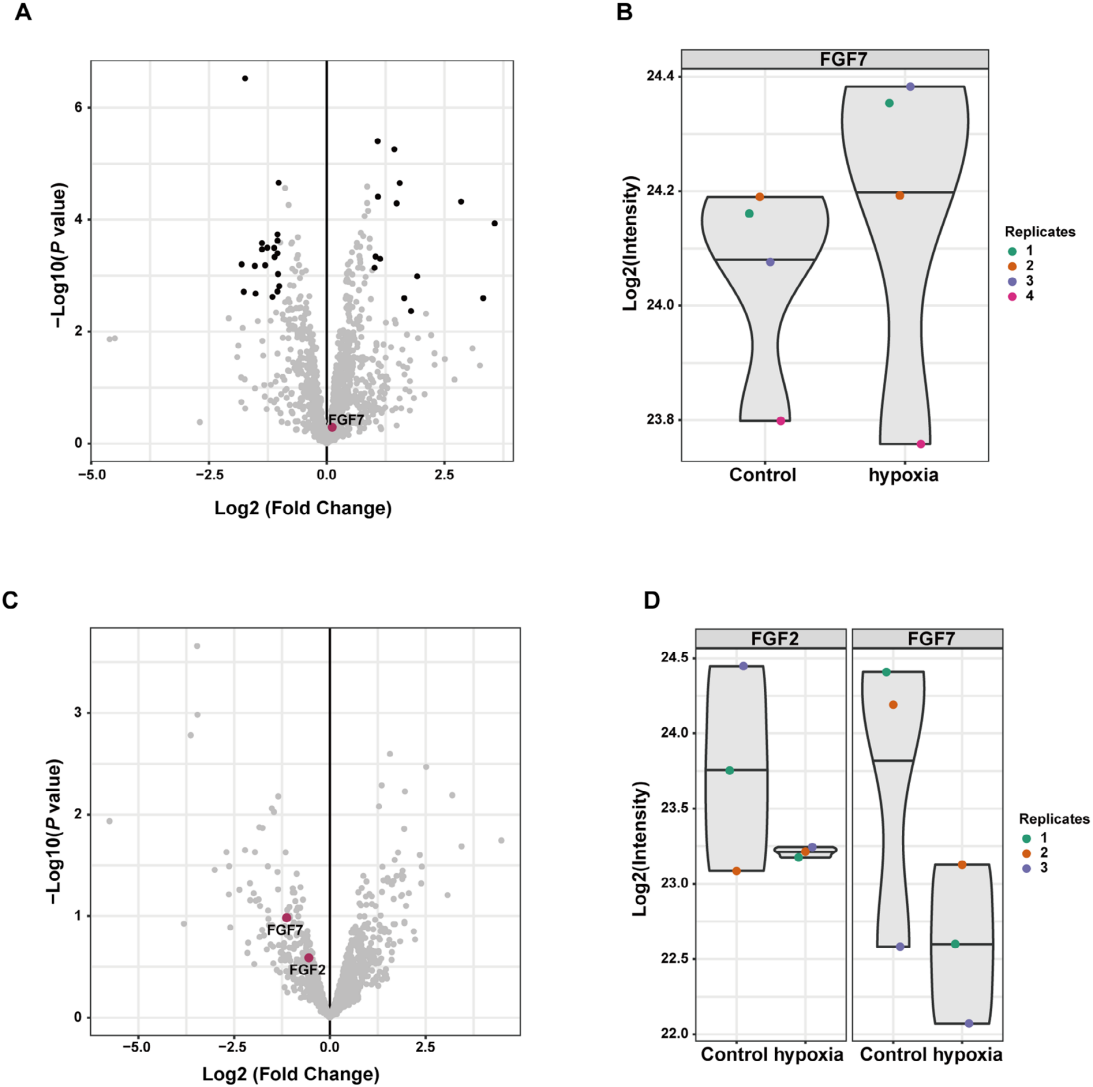
MSCs stably secrete FGF. (A) Volcano plot depicting the difference in protein content in conditioned medium before and after hypoxic induction. (B) Violin plot of FGF7 content in conditioned medium before and after hypoxic treatment. (C) Volcano plot illustrating protein content in MSC‐derived exosomes before and after hypoxic induction. (D) Violin plot of FGF2 and FGF7 content in MSC‐derived exosomes before and after hypoxic treatment.

It is well known that ADSCs can secrete a variety of cytokines, regulating inflammation and promoting tissue regeneration. To investigate whether ADSCs stably secrete angiogenic cytokines under the hypoxic and hyperglycemic conditions of DFUs. we analysed the proteomic dataset PXD052445 from the PRIDE Database (https://www.ebi.ac.uk/pride/). Our analysis revealed that FGF7 levels remained stable before and after hypoxic treatment in mesenchymal stem cell conditioned media (Figure 7A,B). Similarly, no significant changes in FGF2 and FGF7 levels were observed in MSC exosomes before and after hypoxic treatment (Figure 7C,D), suggesting that MSCs maintain stable FGF secretion under hypoxia.

**FIGURE 8:**
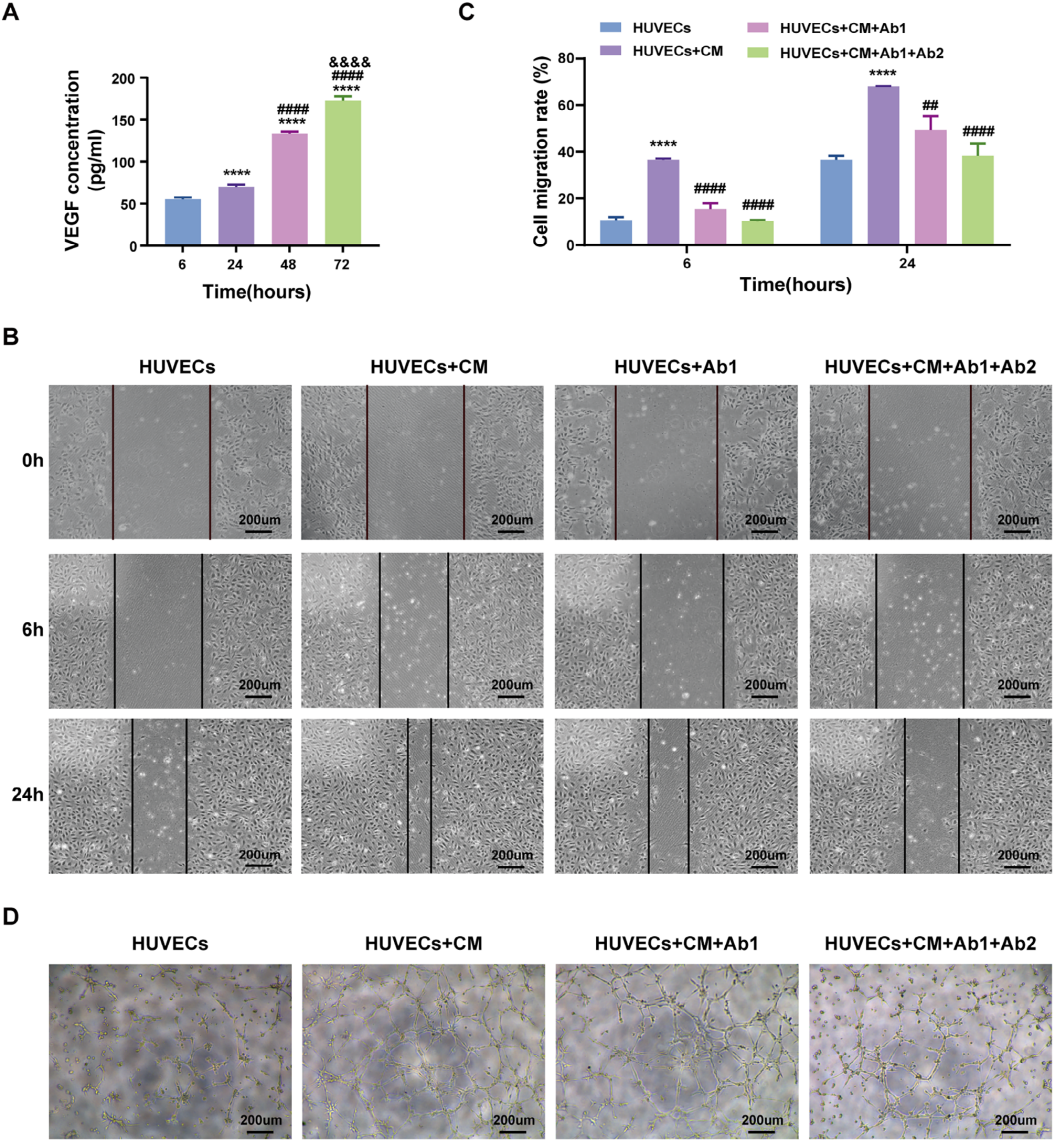
FGF and VEGF secreted by ADSCs promotes angiogenesis. (A) VEGF secretion levels in HUVECs under different conditions measured by ELISA. ****p < 0.0001 vs. 6 h; ^####^ *p* < 0.0001 vs. 24 h; ^&&&&^ *p* < 0.0001 vs. 48 h. (B) Representative images of HUVECs scratch assays at 0, 6, and 24 h in different treatment groups. (C) Quantification of HUVECs migration rates at 6 and 24 h. ****p < 0.0001 vs. HUVECs; ^##^ *p* < 0.01, ^####^ *p* < 0.0001 vs. HUVECs + CM. (D) Representative tube formation assay images of HUVECs cultured under different conditions. Ab1: Neutralising antibody of FGF, Ab2: Neutralising antibody of VEGF, CM: ADSC‐conditioned medium. Data were presented as mean ± SD. Scale bar: 200 μm.

Additionally, we measured the VEGF content in CM using ELISA and found that VEGF secreted by ADSCs steadily increased over time (Figure 8A). To investigate whether FGF and VEGF act synergistically in ADSC‐mediated angiogenesis, we performed neutralisation experiments in HUVECs cultured with CM. Blocking FGF with Ab1 (Cat No. 69024‐1‐Ig, proteintech) significantly reduced HUVEC migration and tube formation. When VEGF was further neutralised with Ab2 (Cat No. 11066‐R010, SinoBiological), the inhibitory effect was even more pronounced (Figure 8B–D).

Taken together, our results indicate that ADSCs promote *FGFR2* expression on one hand and directly secrete FGF on the other, thereby activating the FGF signalling pathway comprehensively and promoting VEGF expression. Concurrently, ADSCs also directly secrete VEGF, exerting a synergistic effect on angiogenesis. Thus, ADSCs exert multifaceted and synergistic therapeutic effects in DFUs.

## 4 Discussion

DFUs are a severe complication of diabetes, characterized by impaired wound healing and a high risk of amputation. These ulcers result from a complex interplay of factors, including neuropathy, ischemia, and infection, all of which contribute to poor clinical outcomes [1, 33]. Among the multifactorial pathogenesis of DFUs, impaired angiogenesis plays a central role in the delayed healing process [34]. Angiogenesis is essential for supplying oxygen, nutrients, and immune cells to the wound site, thereby facilitating tissue repair and regeneration [35]. Chronic hyperglycemia in patients with DFUs induces oxidative stress and inflammation, leading to endothelial cell dysfunction, which in turn disrupts angiogenesis [36]. Additionally, hyperglycemia promotes the accumulation of AGEs, which further impair endothelial cell function by binding to their receptor (RAGE) and activating pro‐inflammatory pathways [37]. Despite significant advances in understanding the role of angiogenesis in DFUs, research on molecular targets that enhance angiogenesis in DFU patients remains relatively limited.

In this study, we identified and validated key molecular targets involved in angiogenesis in DFUs by combining bioinformatics analysis with in vivo and in vitro experiments. Initially, we analysed the DEGs between DFU patients and the control groups using datasets from the GEO database. Given the critical role of angiogenesis in DFUs' wound healing and enrichment analysis indicating suppressed angiogenic signalling in DFUs, we further identified An‐DEGs. Subsequently, key An‐DEGs were identified using the PPI network and machine learning algorithms. Through expression analysis of these genes in the validation sets as well as in vivo and in vitro experiments, we identified *FGFR2* suppression as a central factor contributing to angiogenesis dysfunction in DFUs.

*FGFR2*, a key member of the *FGFR* family, plays a pivotal role in regulating angiogenesis, cell proliferation, and tissue repair [38]. Our study demonstrated that impaired FGF signalling was a critical factor contributing to delayed wound healing. This finding aligns with previous studies highlighting the importance of *FGFR2* in vascular development and repair. For instance, Farooq et al. [28] reported that *FGFR2* activation using an agonistic peptide significantly enhanced angiogenesis and collagen deposition in diabetic wounds, further underscoring its therapeutic potential.

Given the essential role of angiogenesis in wound healing, various therapeutic strategies have been explored to enhance angiogenic potential. Current approaches include growth factor therapy, stem cell therapy, gene therapy and others. Growth factor therapies, such as recombinant human PDGF (PDGF, Becaplermin), have demonstrated efficacy in promoting wound healing, but their clinical application is limited by short half‐lives and high costs [39]. Stem cell therapies, particularly those utilising ADSCs and bone marrow‐derived mesenchymal stem cells (BM‐MSCs), have shown promise in enhancing angiogenesis through the secretion of pro‐angiogenic factors and exosomes [40, 41]. Gene therapy, including VEGF gene delivery and CRISPR‐Cas9‐based approaches, offers the potential for sustained expression of therapeutic proteins; however, challenges related to delivery efficiency and safety remain [42]. Despite their promise, current therapeutic strategies targeting angiogenesis are constrained by limitations such as short half‐lives, unclear molecular mechanisms, and potential safety concerns.

MSCs, particularly ADSCs, have emerged as a promising approach due to their ability to secrete pro‐angiogenic factors and modulate the wound microenvironment [14, 28, 43]. Previous studies have shown that ADSCs exert their pro‐angiogenic effects primarily through the paracrine secretion of cytokines such as VEGF and FGF [44, 45, 46]. This study demonstrated, for the first time, that ADSCs upregulate *FGFR2* expression, thereby activating the FGF‐PI3K/Akt‐HIF‐1α‐VEGF signalling axis, which is essential for promoting angiogenesis and wound healing. Further analysis of public database data revealed that ADSCs not only enhance *FGFR2* expression but also directly secrete FGF, creating a synergistic effect that amplifies downstream signalling activation [47]. This dual mechanism highlights the multifaceted therapeutic effects of ADSCs. Additionally, our previous study found that ADSCs persist in vivo for up to 28 days, with a half‐life significantly longer than that of cytokines such as PDGF, thereby enabling prolonged pro‐angiogenic effects.

Through the bioinformatics analysis and experimental validations conducted in this study, we have elucidated a core molecular mechanism underlying angiogenesis impairment in DFU patients. These findings provide valuable insights for the clinical diagnosis of DFUs and the therapeutic mechanisms of ADSCs. However, there are several limitations in this study. First, the precise molecular mechanisms by which ADSCs promote *FGFR2* expression still require further investigation. Second, while our validation of Fgfr2 was conducted using a DFU mouse model, an ideal chronic wound healing model for DFUs remains lacking. Existing DFU mouse models may not accurately recapitulate the ulcer environment in DFU patients, as untreated wounds in these models typically undergo epithelialization within 14 days. Lastly, wound contraction plays a crucial role in murine wound healing but has a lesser impact on early human wound healing [48]. Therefore, in future studies, we plan to use splinting and are committed to researching more suitable mouse models. To address these limitations, future studies will focus on elucidating the regulatory mechanisms of ADSCs‐induced *FGFR2* expression and developing more physiologically relevant animal models that better mimic human chronic wound healing.

## Author Contributions

**Jing Cao:** conceptualization (equal), data curation (lead), formal analysis (lead), writing – original draft (lead). **Zichao Liu:** data curation (equal), validation (lead). **Wenqiang An:** investigation (equal), methodology (lead). **Xin Zhang** and **Zhujun Li:** methodology (equal). **Lijie Li:** funding acquisition (lead). **Hailian Ji:** funding acquisition (equal). **Sen Zhang:** methodology (equal), validation (equal). **Xiao Long:** resources (equal), supervision (lead). **Yuemei Yang:** conceptualization (equal), project administration (lead), writing – review and editing (equal).

## Ethics Statement

Human adipose‐derived stem cells (ADSCs) used in this study were obtained from discarded liposuction tissue with informed patient consent, following protocols approved by the Ethics Committee of Peking Union Medical College (Approval No.: ZS‐3186; Project Title: Establishment of a Biological Sample Bank for Human Adipose‐Derived Mesenchymal Stem Cells; Approval Date: October 26, 2021).

Ethical approval for the animal experiments was granted by the Ethics Committee of the Chinese Academy of Medical Sciences (Approval No.: 00009780; Project Title: Efficacy Study of Local Injection of Human Adipose‐Derived Mesenchymal Stem Cells for Treating Diabetic Foot Ulcers in Mice; Approval Date: May 5, 2023).

## Conflicts of Interest

The authors declare no conflicts of interest.

## Supporting information


**Appendix S1:** jcmm70942‐sup‐0001‐AppendixS1.docx.

**Appendix S2:** jcmm70942‐sup‐0002‐AppendixS2.docx.

**Appendix S3:** jcmm70942‐sup‐0003‐AppendixS3.csv.

**Appendix S4:** jcmm70942‐sup‐0004‐AppendixS4.csv.

## Data Availability

The data that support the findings of this study are available from the corresponding author upon reasonable request.
