# Supplementary material for: FGFR2 is a Crucial Factor for Adipose‐Derived Mesenchymal Stem Cells in Promoting Diabetic Foot Ulcer Healing Through Angiogenesis
Source: J Cell Mol Med. 2025 Nov 13;29(21):e70942. doi: 10.1111/jcmm.70942 (PMC12613079; doi:10.1111/jcmm.70942)
Supplement: Supplementary file 1 — Appendix S1: jcmm70942‐sup‐0001‐AppendixS1.docx. [file JCMM-29-e70942-s004.docx]

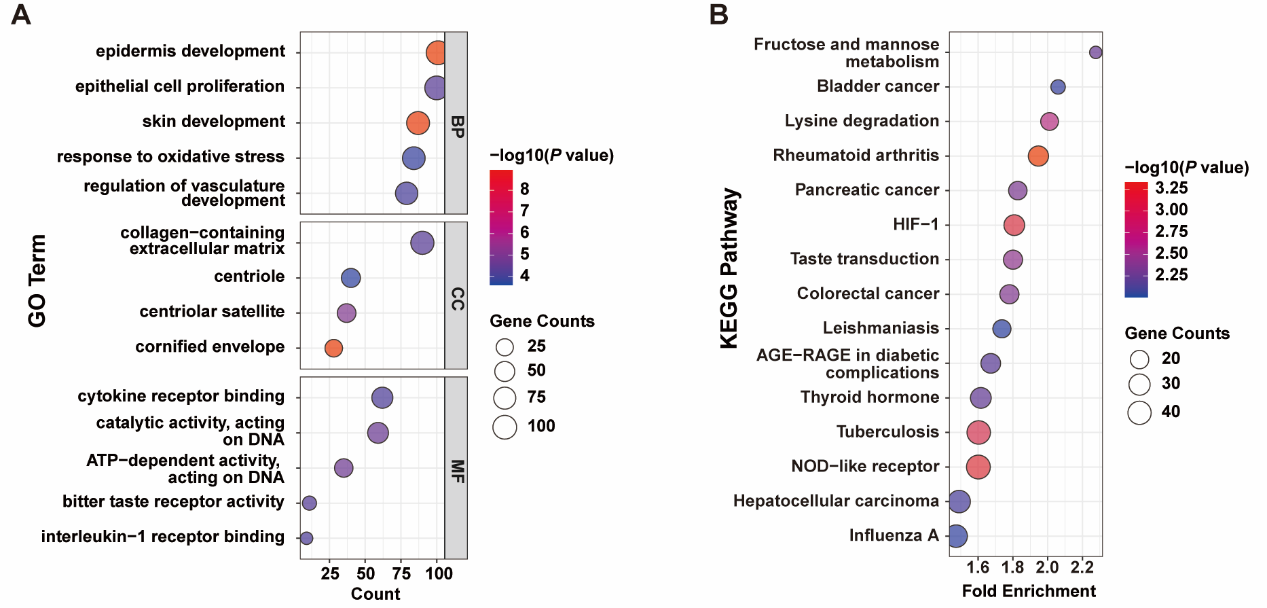


**Figure S1.** Enrichment analysis of dataset GSE80178. **(A)** GO and **(B)** KEGG pathway analysis.


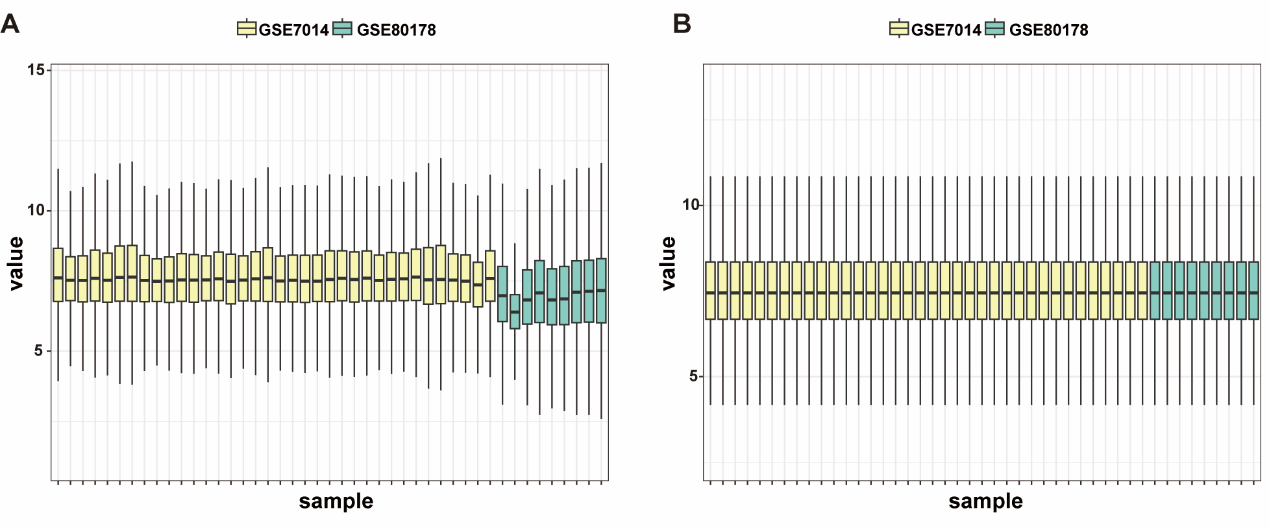


**Figure S2. Gene expression in GSE7014 and GSE80178 before and after batch correction. (A) Before and (B) after of batch correction.**
