# Supplementary material for: FGFR2 is a Crucial Factor for Adipose‐Derived Mesenchymal Stem Cells in Promoting Diabetic Foot Ulcer Healing Through Angiogenesis
Source: J Cell Mol Med. 2025 Nov 13;29(21):e70942. doi: 10.1111/jcmm.70942 (PMC12613079; doi:10.1111/jcmm.70942)
Supplement: Supplementary file 2 — Appendix S2: jcmm70942‐sup‐0002‐AppendixS2.docx. [file JCMM-29-e70942-s002.docx]

(a) (b)


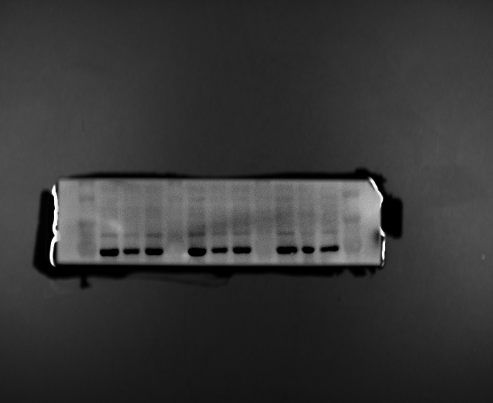

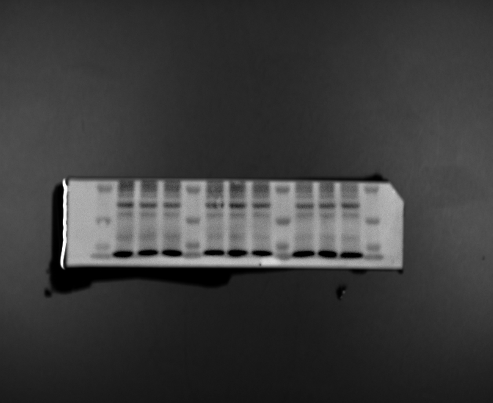


**Figure 5B.** Fgfr2 protein expression before and after ADSCs administration. **(a)** Original full-length uncropped blots of FGFR2 and **(b)** Gapdh from DFU mice. The lanes within the red box represent protein ladder: Control (Untreated mice), Model (DFU mice), and ADSCs (ADSCs treated DFU mice), respectively.

(a) (b)


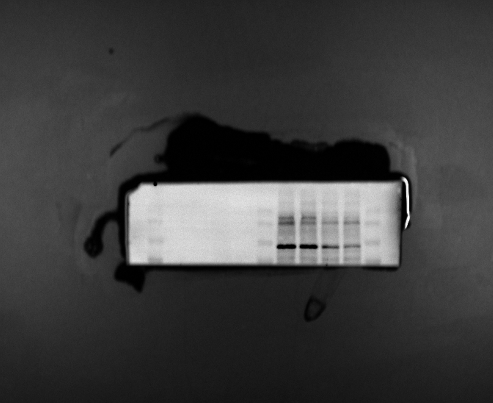

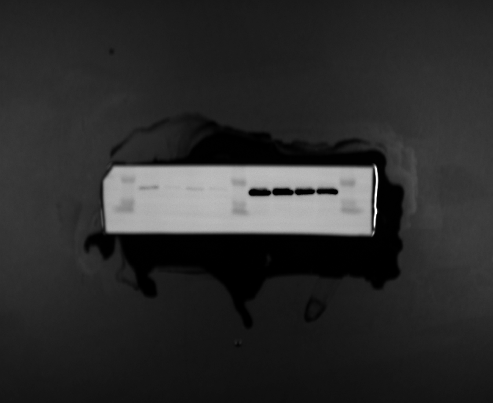


**Figure 6B**. FGFR2 protein expression before and after siRNA transfection. **(a)** Original full-length uncropped blots of FGFR2 and **(b)** β-actin from Human Umbilical Vein Endothelial Cells (HUVECs). The lanes within the red box represent protein ladder: HUVECs, HUVECs + siFGFFR2-1, HUVECs + siFGFFR2-2, HUVECs + siFGFFR2-3, respectively.

(a) (b)


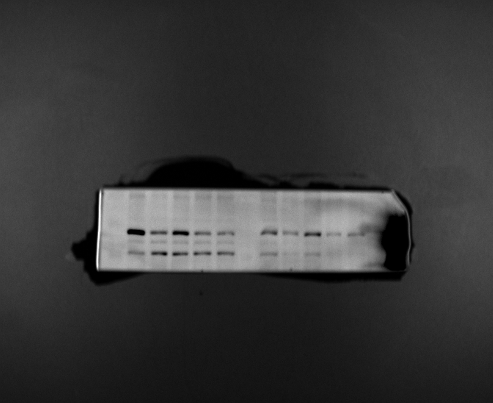

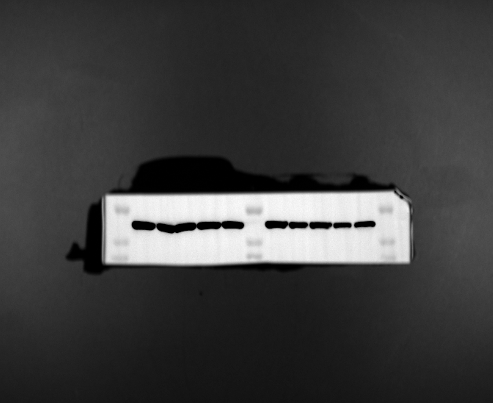


**Figure 6D.** siFGFR2 inhibits the upregulation of FGFR2 by Adipose-derived Stem Cells. **(a)** Original full-length uncropped blots of FGFR2 and **(b)** β-actin from HUVECs. The lanes within the red box represent protein ladder: HUVECs, advanced glycation end-products treated HUVECs (AGE-HUVECs), AGE-HUVECs + ADSCs, AGE-HUVECs + ADSCs + siFGFFR2, respectively.

(a) (b)


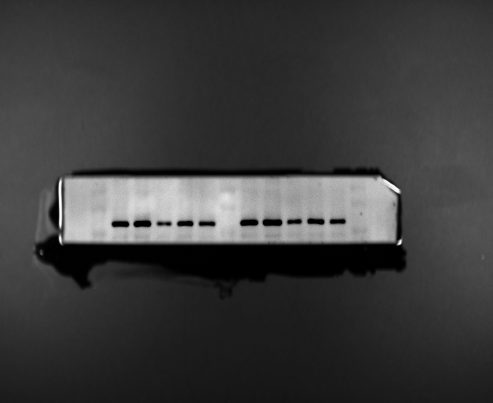

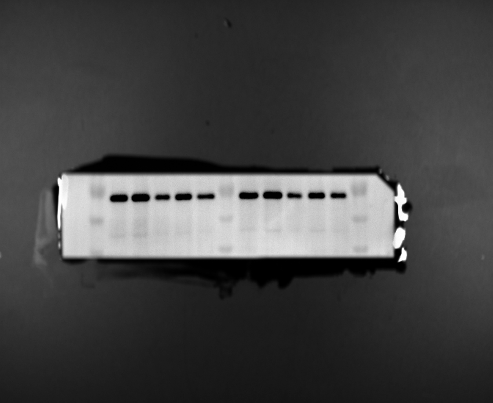


(c) (d)




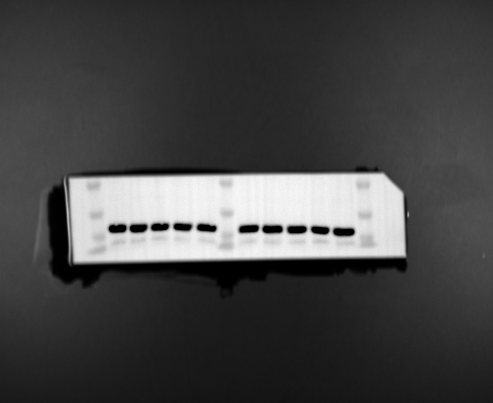


**Figure 6G.** siFGFR2 inhibits the upregulation of PI3K, pAKT, HIF-1α by Adipose-derived Stem Cells. **(a)** Original full-length uncropped blots of PI3K, pAKT, HIF-1α and **(b)** β-actin from HUVECs. The lanes within the red box represent protein ladder: HUVECs, HUVECs + ADSCs, advanced glycation end-products treated HUVECs (AGE-HUVECs), AGE-HUVECs + ADSCs, AGE-HUVECs + ADSCs + siFGFFR2, respectively.
